# Consensus-informed Development of Scoring Systems for Intermediate Laparoscopic Simulation Modules: An ESU Laparoscopic Workgroup Initiative

**DOI:** 10.1016/j.euros.2026.03.014

**Published:** 2026-04-15

**Authors:** Arianna Pischetola, Moises Rodriguez Socarras, Tiago Ribeiro de Oliveira, Silvia García Barreras, Luis E. Ortega Polledo, Patricia J. Zondervan, Bhaskar K. Somani, Evangelos Liatsikos, Domenico Veneziano, Pia Kraft, Pavel Navratil, Juan Gómez Rivas, Willem M. Brinkman

**Affiliations:** a Faculty of Medicine, Campus Bio-Medico University, Rome, Italy; b Instituto de Cirugía Urológica Avanzada (ICUA), Clínica CEMTRO, Madrid, Spain; c Department of Urology, Armed Forces Hospital, Lisbon, Portugal; d Department of Urology, Hospital Universitario Ramón y Cajal, Madrid, Spain; e Department of Urology, Hospital Clínico San Carlos, Madrid, Spain; f Department of Urology, Amsterdam UMC, Cancer Center Amsterdam, University of Amsterdam, Amsterdam, The Netherlands; g University Hospital Southampton, Department of Urology, Southampton, United Kingdom; h Department of Urology, University Hospital of Patras, Patras, Greece; i Department of Urology, Smith Institute for Urology, Northwell Health, New York, NY, USA; j Division of Urology, Department of Surgery, Princess Margaret Cancer Centre, University Health Network, University of Toronto, Toronto, ON, Canada; k Department of Urology, University Hospital Hradec Kralove, Hradec Králové, Czech Republic; l Department of Oncological Urology, University Medical Centrum Utrecht, Utrecht, The Netherlands

**Keywords:** Laparoscopy, Simulation training, Urology education, Clinical competence, Validation study, Benchmarking, Partial nephrectomy, Vascular injuries

## Abstract

**Background and objective:**

Competency-based and standardised surgical training has become essential in urological education. The European School of Urology (ESU) established the Standardisation in Surgical Education initiative to develop a validated, competency-based surgical training. The ESU Laparoscopic Workgroup aims to develop two intermediate-level laparoscopic simulation modules, Partial Nephrectomy (PN) and Major Vessel Injury (MVI), and develop standardised performance metrics and scoring criteria for the intermediate laparoscopic ESU curriculum.

**Design, setting and participants:**

Modules were evaluated during hands-on training courses across multiple European congresses. PN was completed by 27 participants (four experts, 23 residents); MVI by 41 participants (29 experts and 12 residents). Predefined performance parameters were derived from a previously published Delphi consensus defining the ESU Laparoscopic Urological Skills 2 curriculum and included procedure time, tissue handling, technical precision, completeness of resection/closure, haemostasis, and functional outcome.

**Outcome measurements and statistical analysis:**

Completion times were assessed using the Mann-Whitney U test for continuous variables. Qualitative performance parameters were recorded as criterion-referenced educational indicators and summarised descriptively. Observed performance patterns, together with expert consensus, were used to inform the development of candidate scoring frameworks.

**Results:**

Experts completed both modules in shorter median times compared with residents, for both modules (PN: 21:36 [interquartile range {IQR} 17:49–26:21] min vs 42:00 [IQR 36:35–43:26] min, *p* = 0.001; MVI: 5:24 [IQR 4:57–8:04] min vs 16:48 [IQR 11:12–27:15] min, *p* < 0.001). Qualitative categorical parameters (tissue handling, resection completeness, and haemostasis) showed descriptive patterns favouring expert participants and were reported narratively. Informed by these observed performance patterns and the underlying Delphi consensus, structured, domain-based candidate scoring frameworks were proposed for both modules to support future validation and curriculum implementation.

**Conclusions:**

This study supports the feasibility and educational relevance of two intermediate laparoscopic simulation modules within the ESU LUS2 curriculum. Building on a previously established Delphi consensus and informed by observed performance patterns, we propose preliminary, consensus-informed scoring frameworks intended to guide structured assessment and feedback. These findings should be regarded as a foundational step toward future validation and refinement in larger cohorts prior to formal implementation.

## Take Home Message

This study established criteria for two European School of Urology (ESU) intermediate laparoscopic simulation modules: Partial Nephrectomy and Major Vessel Injury. Their standardized scoring systems establish benchmarks for assessing intermediate-level laparoscopic proficiency within the ESU curriculum.

ADVANCING PRACTICE **What does this study add?**

This study establishes criteria for intermediate-level laparoscopic simulation modules for partial nephrectomy and major vessel injury within the European School of Urology curriculum. It suggests a scoring system and competency thresholds. These modules bridge the gap between basic and advanced laparoscopic training, offering a reproducible framework for certification and structured skill development across Europe.

**Patient Summary**

We developed and tested two new simulation modules to help surgeons practice complex laparoscopic skills in a safe environment. Our study showed that these training tools can distinguish between different levels of surgical experience. These modules provide a standardized way to ensure surgeons reach a high level of proficiency before performing surgery on patients.

## 1 Introduction

Competency-based surgical training has become an essential component of modern urological education. The European School of Urology (ESU), guided by the European Association of Urology (EAU), has progressively built a global standard for structured and validated training in urology [1]. Through the Standardisation in Surgical Education (SISE) initiative, curricula are developed via cognitive task analysis, Delphi consensus, and iterative validation through simulation-based testing [2]. Recent developments in laparoscopic urology training include advances in virtual reality (VR), three-dimensional printed organs and perfused ex vivo simulations [3], [4], [5], [6].

Within this framework, the Laparoscopic Workgroup focuses on modular training across basic, intermediate, and advanced levels. While basic skills are established through programmes such as the European Basic Laparoscopic Urological Skills (E-BLUS) [7], intermediate modules aim to bridge technical complexity and real-world application [8].

Intermediate and advanced laparoscopic skills are challenging to acquire in the operating room alone. Training therefore increasingly relies on simulation to ensure proficiency before patient operations, optimising outcomes [9]. Simulation-based modules provide risk-free hands-on practice in standardised environments [10]. Partial Nephrectomy (PN) and Major Vessel Injury (MVI) represent prime examples of high complexity and high-risk scenarios where simulations offer substantial value. PN models renal resection and reconstruction, assessing precision, spatial awareness, and haemostasis. MVI reproduces vascular repair under simulated bleeding, emphasising rapid decision-making, tissue handling, and suturing under pressure.

Based on the European intermediate LUS curriculum we aim to translate the previously established Delphi consensus of the LUS2 curriculum into measurable performance domains and to propose preliminary, consensus-informed scoring frameworks for two intermediate laparoscopic simulation modules to support future validation and curriculum implementation.

## 2 Materials and methods

### 2.1 Study design and context

This study was conducted by the ESU Laparoscopic Workgroup, following the SISE validation process [2]. Data were collected across multiple ESU and EAU Hands-on Training events **(**Appendix 1**)**, with both experts and residents performing standardised tasks under supervision.

Both modules were designed to explore expected performance patterns across experience levels and to inform the development of candidate performance benchmarks for certification. These models were selected because they allow structured assessment of key technical domains relevant to intermediate procedures, including tissue handling, suturing precision, haemostatic control, and procedural flow. In addition, both models incorporate life-like materials and anatomically representative configurations that provide realistic tactile feedback and spatial relationships, enabling meaningful observation of technical execution under standardised conditions. At the time of data collection, these models were selected because these were the models fully developed for consistent use at the time of data collection, whereas other prototypes were still undergoing refinement.

### 2.2 Objective

The primary objective of this study was to identify and describe predefined performance parameters that could inform the development of a standardised assessment framework for the ESU intermediate laparoscopic curriculum. The secondary objective was to explore whether these parameters demonstrate consistent and meaningful variation across surgeons with differing levels of operative experience, thereby supporting their relevance and educational relevance to inform future benchmarking and curriculum implementation.

### 2.3 Simulation models and task descriptions

#### 2.3.1 PN

The PN simulation replicates the critical intraoperative steps of tumour excision and renal reconstruction performed during minimally invasive nephron-sparing surgery. The exercise is conducted within a standardised laparoscopic training box equipped with a high-definition camera and a fixed port configuration (Appendix 2).

#### 2.3.2 MVI

MVI simulation models the management of a sudden intraoperative vascular injury and subsequent repair under laparoscopic conditions. The exercise is designed to reproduce the essential steps of exposure, bleeding control, and primary suture repair of a major vessel, assessing accurate intracorporeal suturing under time constraints while maintaining haemostasis and tissue integrity (Appendix 3).

### 2.4 Data collection

Data collection was conducted prospectively between September 2023 and September 2025 during the European congresses and affiliated hands-on teaching events, including urologists in formal training as the “resident” cohort and, as the “experts” (fully trained urologists who have completed specialist training and have substantial experience in laparoscopic procedures) who participated in the training events as tutors or faculty. All exercises were performed under standardised conditions and supervised by certified ESU tutors. Each participant self-declared their level of expertise (expert, resident, or undefined). All performances were observed and documented in real-time by trained assessors using predefined, a priori outcome parameters embedded in the standardised and training framework and subsequently compiled for exploratory analysis of performance patterns across levels of experience. Qualitative variables were defined as criterion-referenced educational indicators.

For the PN module, the following variables were recorded:

• Procedure time: total duration from the first incision into the parenchymal model to the placement of the final Hem-o-lock clip or declaration of closure.
• Tissue handling: evaluation of precision, steadiness, and avoidance of excessive traction or tearing of the synthetic parenchyma.
• Completeness of tumour resection: confirmation that the artificial tumour was entirely excised with adequate simulated margins.
• Quality of renorrhaphy: assessment of closure technique and suture spacing.
• Intraoperative errors: documentation of suture breakage, excessive manipulation, or tearing of the parenchyma.

For the MVI module, the parameters analysed included:

• Procedure time: total duration from the onset of simulated bleeding (opening of perfusion) to the final removal of the needle after repair.
• Tissue handling: assessment of atraumatic manipulation, precision of grasping, and needle control on the synthetic vessel wall.
• Suturing technique: evaluation of stitch placement, symmetry, and adequacy of closure under simulated perfusion.
• Bleeding control: confirmation of immediate and sustained haemostasis with no leakage under physiologic flow for at least 10 s.
• Simulated blood loss: recorded volumetrically during the exercise, with a maximum allowable loss of 3 l defining task failure.
• Integrity of repair: observation for any tearing, additional vessel damage, or residual leakage following task completion.

Additionally, data were collected through model assessment questionnaires completed by each participant immediately after task performance. These questionnaires aimed to evaluate participants’ subjective perceptions of model realism, educational value, and relevance to clinical practice. Each item was rated on a 1–10 numerical scale, providing quantitative measures of perceived anatomical accuracy, tactile feedback, visual realism, and the model’s ability to replicate intraoperative challenges. These subjective evaluations accompanied the objective performance data, providing complementary information on perceived realism, educational value, and relevance to clinical practice (Appendix 4).

All tasks were supervised and assessed by ESU faculty members of the ESU Laparoscopic Workgroup, comprising experienced laparoscopic surgeons in the field, involved in the development of the curriculum. Their role included ensuring standardised task execution, performing real-time assessment using predefined criteria, and prospectively collecting performance data. The same group subsequently contributed to data analysis and development of the proposed scoring frameworks.

### 2.5 Statistical analysis

Descriptive statistics were reported with median and interquartile ranges (25^th^–75^th^). Completion times were analysed as a continuous variable and compared between experts and residents using the Mann-Whitney U test to explore expected performance patterns across experience levels. A *p* value <0.05 was considered statistically significant for completion time comparisons only.

Qualitative performance parameters and categorical outcomes (eg, bleeding, exceeding predefined time limits) were collected prospectively as categorical, criterion-referenced variables based on predefined safety and competency thresholds within the ESU assessment framework, rather than as continuous measurements, and thus summarised descriptively only. No inferential statistical testing was performed for these variables. Statistical analyses were exploratory in nature and were not intended to validate scoring systems.

All analyses were conducted using the latest versions of Microsoft Excel (Microsoft Corp., Redmond, WA) and IBM SPSS Statistics Version 31 (IBM Corp., Armonk, NY). Participants initially labelled as “undefined” were reassigned to either the expert or resident cohort based on available background information, with no changes made to performance data.

## 3 Results

### 3.1 PN

A total of 27 participants completed the PN module, including four experts and 23 residents. Median completion time for experts was 21:36 (interquartile range [IQR] 17:49–26:21) min, whereas for residents it was 42:00 (IQR 36:35–43:26) min; Mann-Whitney U = 30, *p* = 0.001 (Table 1).

**Table 1:** Procedure time by expertise level for partial nephrectomy and major vessel injury models

**Model**	***Expertise level***	***N***	**Median, IQR (min:s) (25–75)**
**Partial nephrectomy**	Expert	4	21:36 (17:49–26:21)
	Resident	23	42:00 (36:35–43:26)
**Major vessel injury**	Expert	29	5:24 (4:57–8:04)
	Resident	12	16:48 (11:12–27:15)

The table depicts the descriptive statistics (median, IQR; 25th–75th) of TIME for Experts and Residents. All values are reported in mins.

IQR = interquartile range; min = minutes, s = seconds.

Qualitative performance parameters, including tissue handling, resection completeness, and haemostasis, were assessed by direct observation during task execution. Experts demonstrated smoother execution with fewer corrective manoeuvres during renorrhaphy.

### 3.2 MVI

A total of 41 participants performed the MVI module (29 experts and 12 residents) performed the MVI task. The median completion time experts were 5:24 (4:57–8:04) min, whereas for residents 16:48 (11:12–27:15) min; Mann-Whitney U = 50.5, *p* < 0.001(Table 1).

Experts were observed to achieve watertight closures with minimal simulated blood loss (<1 l), whereas residents more frequently exceeded the predefined 20-min time limit or demonstrated residual leakage or simulated blood loss exceeding 3 l.

### 3.3 Development of standardised scoring systems

Following completion of data collection, the ESU Laparoscopic Workgroup conducted a post hoc evaluation of all recorded parameters to inform the development of candidate scoring frameworks for the PN and MVI modules. This process integrated observed performance patterns from the current dataset, identified by the ESU Laparoscopic Workgroup, with the previously established Delphi consensus process, which integrated observed data trends with collective clinical experience. The resulting parameters should be regarded as expert-derived, consensus-informed candidate determinants of procedural performance, intended to support structured assessment and guide future validation efforts [8].

For the PN module, the Workgroup identified procedure completion time, renorrhaphy quality, tumour resection completeness, and tissue handling precision as key domains relevant to intermediate-level performance. These domains were translated into a four-domain scoring framework, with each domain scored on a 4-point Likert scale (Excellent–Poor).

Provisional competency criteria were defined to reflect expected technical adequacy at the intermediate training level, including completion of the procedure within 40 min, watertight closure without leakage, gentle and controlled tissue handling, and complete tumour excision with adequate simulated margins. Failure to meet any of these standards, including overt model tearing or incomplete closure, resulted in a downgraded score. The passing threshold was proposed at ≥10 points (≥60%), to indicate overall technical adequacy at the intermediate level.

For the MVI module, procedure completion time, bleeding control, suture integrity, and tissue handling were identified as core assessment domains based on expert consensus and observed task execution characteristics. These domains informed the development of a five-domain scoring system, again using a 4-point Likert scale.

Proposed performance targets included completion within 20 min, achievement of immediate and sustained haemostasis with no visible leakage for at least 10 s, blood loss <3 l, and maintenance of atraumatic tissue manipulation. Excessive blood loss or vessel tearing constituted automatic failure. A provisional passing score of ≥12 points (≥60%) was selected to align with ESU intermediate-level competency benchmarks.

Together, these proposed scoring frameworks provide a structured approach to performance assessment for the PN and MVI modules and are intended to serve as foundation for future validation, reliability testing, and refinement prior to formal integration into the ESU LUS2 curricula (Table 2, Table 3).

**Table 2:** Standardized scoring system for partial nephrectomy

Domain	Excellent (4 pt)	Good (3 pt)	Fair (2 pt)	Poor (1 pt)
Procedure time	≤40 min	41–46 min	47–52 min	≥52 min
Tumour resection	Complete, clear margins	Complete, minor uncertainty	Difficult plane correction	Incomplete resection
Renorrhaphy	Watertight, haemostatic	Minor issues, watertight	Gapping/poor spacing	Ineffective closure
Tissue handling	Atraumatic	Occasional roughness	Frequent roughness	Tissue damage

pt = points, min = minutes.

**Domains:** 4.

**Each domain:** 1 to 4 points.

**Total:** 4–16 points.

**Passing score:** ≥10 points (≥60%).

**Table 3:** Standardized scoring system for major vessel injury

Domain	Excellent (4 pt)	Good (3 pt)	Fair (2 pt)	Poor (1 pt)
Procedure time	≤20 min	21–25 min	26–30 min	>30 min
Bleeding control	Immediate seal, no bleeding ≥10 s	Minor bleeding, controlled	Repeated attempts	Persistent/late bleeding
Blood loss	<1 l	1–2 l	2–3 l	≥3 l
Tissue handling	Atraumatic	Occasional force	Frequent roughness	Vessel tear
Suture quality	Watertight, correct technique	Minor issue	Multiple issues	Leak or iatrogenic damage

pt = points, min = minutes.

**Domains:** 5.

**Each domain:** 1 to 4 points.

**Total:** 5–20 points.

**Passing score:** ≥12 points (≥60%).

Detailed task-specific recommendations outlining best practices and common technical pitfalls (“Do’s and Don’ts”) for both modules are provided in Supplementary Tables 4 and 5.

## 4 Discussion

The present study presents as a development step in the implementation of the ESU intermediate laparoscopic curriculum, focusing on the application of two simulation models: PN and MVI. Differences in procedure completion time between expert and resident participants were consistently observed across both modules, reflecting expected performance patterns associated with differing levels of laparoscopic experience, Qualitative aspects of technical execution, including tissue handling and closure quality, showed descriptive trends favouring expert performance but were not subjected to inferential statistical analysis. Together, these findings support the educational relevance and feasibility of the PN and MVI modules as components of intermediate laparoscopic skills training and provide the empirical context for the development of structured, consensus-informed assessment frameworks.

A persistent challenge in surgical education is bridging the gap between simulation-based skill acquisition and the ability to perform safely and effectively in real clinical settings. While programmes such as the E-BLUS establish fundamental laparoscopic dexterity, they do not necessarily ensure procedural competence in complex operative scenarios [7]. True procedural proficiency requires integration of technical precision, spatial awareness, and decision-making capacities that cannot be fully developed through basic training alone. The introduction of structured intermediate modules aims to address this unmet need by offering a structured, competency-based progression training that replicates complex procedures within a standardised and risk-free environment. This approach allows trainees to refine both psychomotor and cognitive skills, supporting a necessary step to real-world operative responsibility.

Another educational consideration is the transition from part-task to whole-task practice. Traditional laparoscopic curricula, such as E-BLUS, deconstruct procedures into discrete technical components that are practised in isolation before integration. While this segmentation supports early skill acquisition, it can limit the ability to transfer integrated performance to complex operations. Whole-task practice, by contrast, involves performing an entire procedure from start to finish, maintaining the cognitive, procedural, and ergonomic context required for fluent execution. Although more demanding, this approach fosters coordination of perceptual, decision-making, and motor skills and better mirrors real operative flow [11]. In this respect, the PN model represents an important step toward whole-task simulation in urology, as it captures sequential stages of tumour excision, renorrhaphy, and haemostatic control within a single operative scenario. Such comprehensive models may enhance contextual learning, promote skill integration, and accelerate readiness for real surgical environments.

Simulation-based surgical education has proven effective in improving psychomotor skills, self-confidence, and operative outcomes while minimising patient risk [9], [12]. Prior studies have shown that criterion-based training, where learners progress upon demonstrated competency, through consequential training goals, rather than fixed exposure time, enhances motivation and overall efficiency [13], [14]. In this study, the use of predefined pass thresholds within proposed scoring frameworks reflects this educational approach and supports standardised feedback and transparent performance expectations, consistent with the SISE framework.

Intermediate and advanced laparoscopic training particularly benefits from physical box-trainer models that provide realistic haptic feedback (the combination of tactile and kinaesthetic feedback), an element still superior to many VR systems [15]. Tactile feedback has been shown to play a crucial role in the early stages of skill acquisition by accelerating the initial portion of the learning curve and improving the overall effectiveness of laparoscopic training [16]. Simulations that incorporate realistic haptic elements are therefore assumed to produce superior training outcomes and promote a more reliable transfer of technical skills to the clinical setting. Several studies have emphasised that tactile feedback plays a critical role in enhancing depth awareness, force modulation, and spatial coordination during minimally invasive procedures, aspects that remain limited in current VR platforms [4], [10], [15]. The silicone-based PN and perfused MVI models used in this study successfully replicate the biomechanical resistance and visual cues of living tissue, providing a high-fidelity training environment and enabling structured observation of technical performance within intermediate laparoscopic simulation.

This study contributes to the growing emphasis on criterion-based training as a foundation for standardised assessment and effective feedback in laparoscopic education. The domain-based scoring framework developed here provides a structured approach that prioritises qualitative aspects of performance over completion time, aligning with the shift toward competency-based education [15]. While operative duration remains an important indicator of procedural efficiency, the inclusion of multidimensional qualitative metrics ensures that training outcomes more accurately reflect real-life surgical practice. Previous work suggests that this approach can enhance learning efficiency, skill retention, and training outcomes while reducing total training time without compromising performance quality [13]. Even in the era of robotic-assisted surgery, proficiency in conventional laparoscopic techniques remains relevant, since studies demonstrate that laparoscopic training improves performance on robotic platforms, facilitating smoother adoption, maintaining better technical control, and preserving a broader skill set. Additionally, many centres continue to favour laparoscopic surgery due to the cost and availability constraints associated with robotic platforms [17], [18]. Within this context, the proposed scoring frameworks are designed to support structured feedback and inform the future development of unified, criterion-based assessment strategies across ESU training programmes.

This study has several limitations. The limited number of experts cohort, mainly due to the conference setting which is required to ensure a standardised testing environment, restricts the generalisability of effect size estimates and limits extrapolation of the proposed performance thresholds to the broader population of practicing urologists. However, the primary objective of the study was not population-level inference, but to evaluate whether predefined performance metrics, particularly qualitative indicators of technical execution, behave consistently with established principles of surgical expertise. In this context, the emphasis on quality-related parameters rather than speed alone strengthens the interpretability and relevance of the findings within a competency-based assessment framework. Additionally, although data was gathered across multiple conferences, the structured hands-on format and strict time and equipment constraints limited the number of participants that could be enrolled without compromising standardised task condition. Furthermore, as with all simulation-based studies, these models cannot fully reproduce the complexity of real operative conditions, and the long-term transfer of acquired skills to clinical performance remains to be established. Given the exploratory nature of the analyses, particularly for secondary outcomes, the findings should be interpreted with appropriate caution.

Several aspects still require refinement. Future work should aim to expand expert participation, increase sample sizes, and evaluate these modules across broader training environments to improve generalisability. The findings should therefore be interpreted as supporting the use of these modules for structured benchmarking and curriculum development, rather than as definitive validation across all training environments or expert populations. Additional studies assessing reliability, learning curves, and transfer to clinical performance are needed to strengthen the evidence base. The absence of a standardised method for assessing hands-on models highlights the need for a unified framework across ESU curricula. Looking ahead, the integration of artificial intelligence-based assessment tools could further enhance the objectivity and scalability of laparoscopic training. Automated analysis of motion patterns and instrument dexterity may provide real-time, data-driven feedback, enabling personalised performance evaluation and continuous skill optimisation.

## 5 Conclusions

This study presents the feasibility of implementing two ESU intermediate laparoscopic simulation modules for PN and MVI within the ESU LUS2 curriculum, in alignment with the SISE framework. Observed differences in procedure completion time between expert and resident participants, together with descriptive trends in technical execution, support the educational relevance of these models for intermediate skills training.

Building on a previously established Delphi consensus and informed by observed performance patterns, this work proposes structured, domain-based scoring frameworks for both modules to support standardised assessment and feedback at the intermediate training level. These scoring systems should be regarded as developmental tools intended to operationalise curriculum objectives and guide future validation, reliability testing, and refinement prior to formal implementation.

  ***Author contributions:*** Arianna Pischetola had full access to all the data in the study and takes responsibility for the integrity of the data and the accuracy of the data analysis.

  *Study concept and design:* Somani, Liatsikos, Veneziano, Rivas.

*Acquisition of data:* Pischetola, Brinkman, de Oliveira, Barreras, Polledo.

*Analysis and interpretation of data:* Pischetola, Socarras, Barreras, Polledo.

*Drafting of the manuscript:* Pischetola, Brinkman, Socarras.

*Critical revision of the manuscript for important intellectual content:* Socarras, Barreras, Polledo, Zondervan, Kraft, Navratil, Rivas.

*Statistical analysis:* Pischetola.

*Obtaining funding:* None.

*Administrative, technical, or material support:* None.

*Supervision:* Rivas.

*Other* (specify)*:* None.

  ***Financial disclosures:*** Arianna Pischetola certifies that all conflicts of interest, including specific financial interests and relationships and affiliations relevant to the subject matter or materials discussed in the manuscript (eg, employment/affiliation, grants or funding, consultancies, honoraria, stock ownership or options, expert testimony, royalties, or patents filed, received, or pending), are the following: None.

  ***Acknowledgment:*** None.
