# Supplementary material for: Consensus-informed Development of Scoring Systems for Intermediate Laparoscopic Simulation Modules: An ESU Laparoscopic Workgroup Initiative
Source: Eur Urol Open Sci. 2026 Apr 15;87:100–6. doi: 10.1016/j.euros.2026.03.014 (PMC13101638; doi:10.1016/j.euros.2026.03.014)
Supplement: Supplementary Data 1 [file mmc3.docx]

**Appendix 1 – Hans-on Training Events**

- 2024 Annual European Association of Urology Congress
- 2025 Annual European Association of Urology Congress
- 2023 European Urology Residents Education Programme
- 2024 European Urology Residents Education Programme
- 2025 European Urology Residents Education Programme
- Human Cadaveric Advanced Laparoscopic Urology Course
