# Supplementary material for: Consensus-informed Development of Scoring Systems for Intermediate Laparoscopic Simulation Modules: An ESU Laparoscopic Workgroup Initiative
Source: Eur Urol Open Sci. 2026 Apr 15;87:100–6. doi: 10.1016/j.euros.2026.03.014 (PMC13101638; doi:10.1016/j.euros.2026.03.014)
Supplement: Supplementary Data 2 [file mmc4.docx]

**Appendix 2 – Description of Partial Nephrectomy Task and Model**

The PN model is produced using a process known as casting or mold casting. 3D printing technology (specifically FDM - Fused Deposition Modeling) is used to manufacture several molds that are subsequently filled with silicone through successive casting steps. Within its internal structure, the model incorporates a network of small tubes that replicate the kidney’s vascular architecture. The silicone material is combined with water-based gels, similar to ultrasound gel, to reduce hardness and provide conductive properties **(Figures 1 and 2).**

**
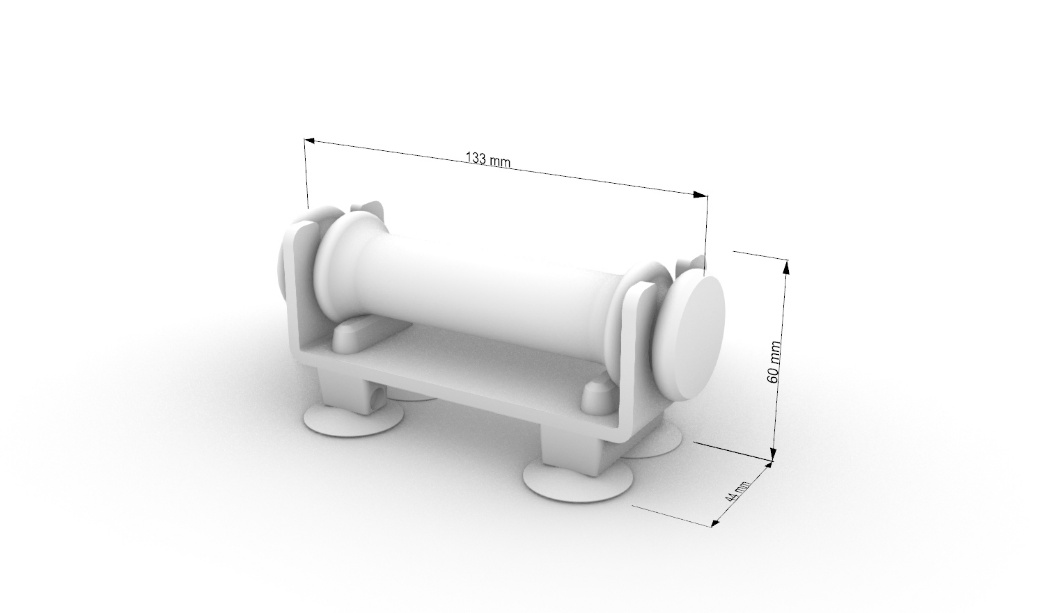
**


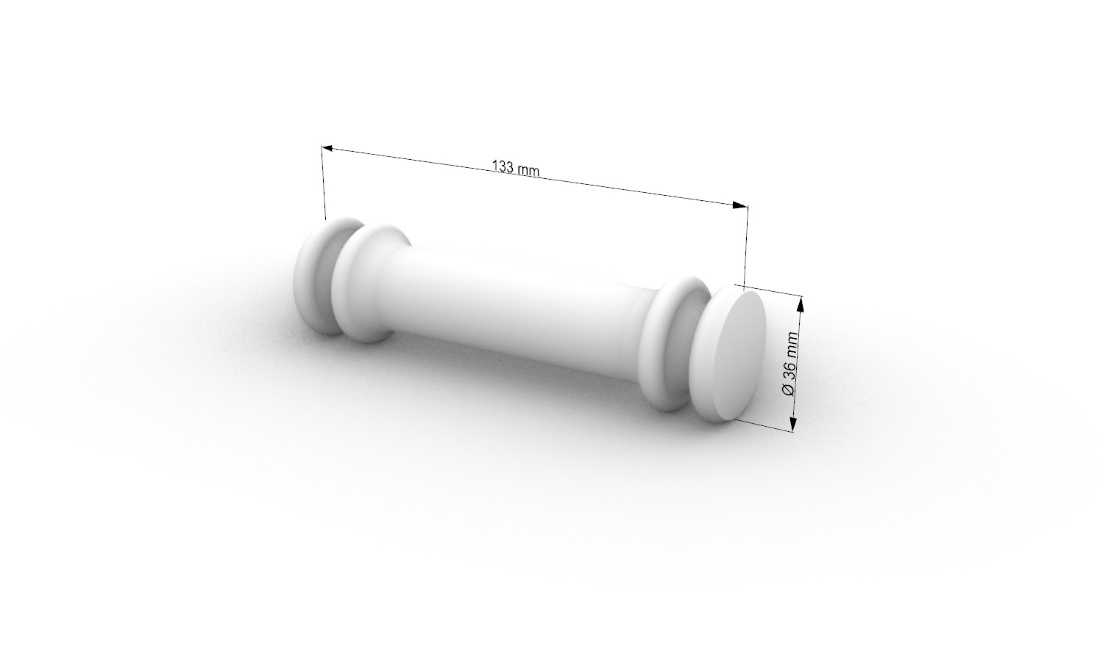


Participants perform the task using either two needle-holders or one needle-holder and a grasper, depending on their preferred bimanual technique. The choice of suture material (monofilament PDS or multifilament Vicryl), suture configuration (running or interrupted), and suture length is left to operator preference. A set of Hem-o-lok clips is available to secure sutures at appropriate steps of the closure.

The task begins once the participant declares readiness. Timing starts at first incision into the parenchyma. The participant must perform a complete tumour resection with adequate safety margins, adhering to the oncological principles of partial nephrectomy. The resection phase is followed by two-layer renorrhaphy, designed to replicate intraoperative reconstruction.

Inner renorrhaphy addresses the collecting system and the deep parenchymal layers while the outer renorrhaphy restores cortical approximation. Sutures are introduced into the field via trocar access and manipulated laparoscopically under direct vision. The use of Hem-o-lok clips at the suture ends simulates parenchymal anchoring and tension control as used in clinical practice.

Throughout the procedure, the participant should demonstrate atraumatic tissue handling, precise needle orientation, and controlled suture tension. Excessive manipulation, tearing of the model tissue, or failure to achieve closure integrity are considered technical errors.

Task completion is defined by the placement of the final Hem-o-lok clip or participant’s formal declaration of closure. The end time is recorded at this point. The exercise therefore evaluates the participant’s ability to perform tumour resection, achieve effective renorrhaphy, and maintain surgical economy and precision within a time-limited environment.
