# Supplementary material for: Consensus-informed Development of Scoring Systems for Intermediate Laparoscopic Simulation Modules: An ESU Laparoscopic Workgroup Initiative
Source: Eur Urol Open Sci. 2026 Apr 15;87:100–6. doi: 10.1016/j.euros.2026.03.014 (PMC13101638; doi:10.1016/j.euros.2026.03.014)
Supplement: Supplementary Data 3 [file mmc5.docx]

**Appendix 3 – Description of Major Vessel Injury Task and Model**

Prior to the exercise, the tutor prepares the model, produced with the same FDM technology, by performing a standardized 5 mm vertical incision at the midpoint of a 133 mm synthetic vessel with a 36 mm diameter **(Figure 3 and 4)**. The incision is verified in front of the participant to ensure consistency of defect size and location. The model is then fixed securely inside the laparoscopic box trainer and connected to a closed perfusion circuit containing 3 L of synthetic blood, positioned 10 cm above the model to provide physiologic flow. This setup ensures reproducible pressure and realistic bleeding dynamics once perfusion is initiated.


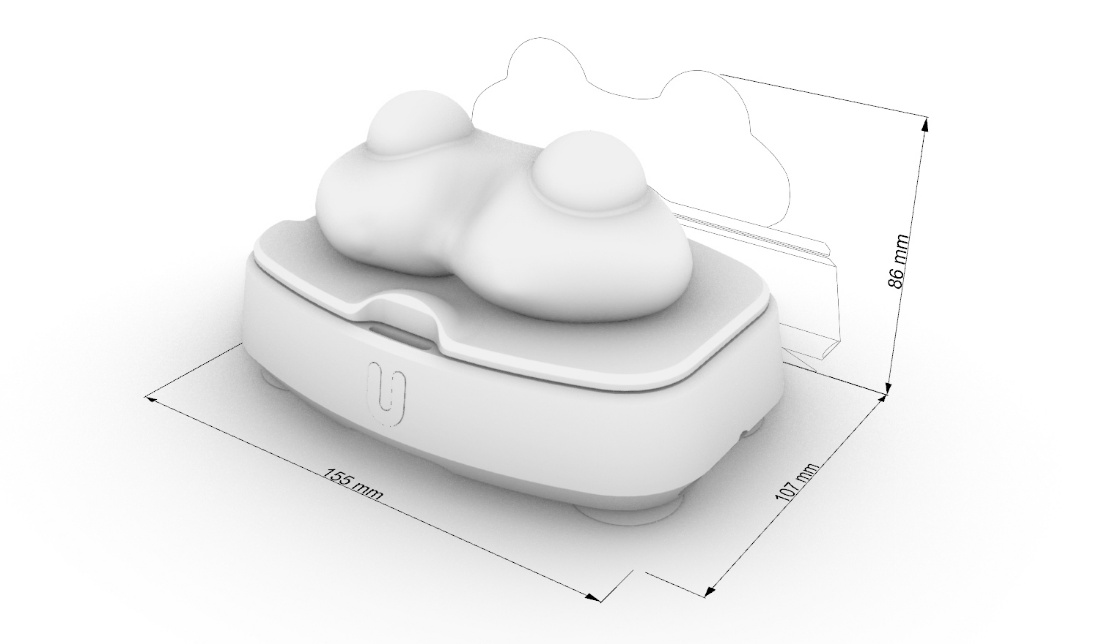


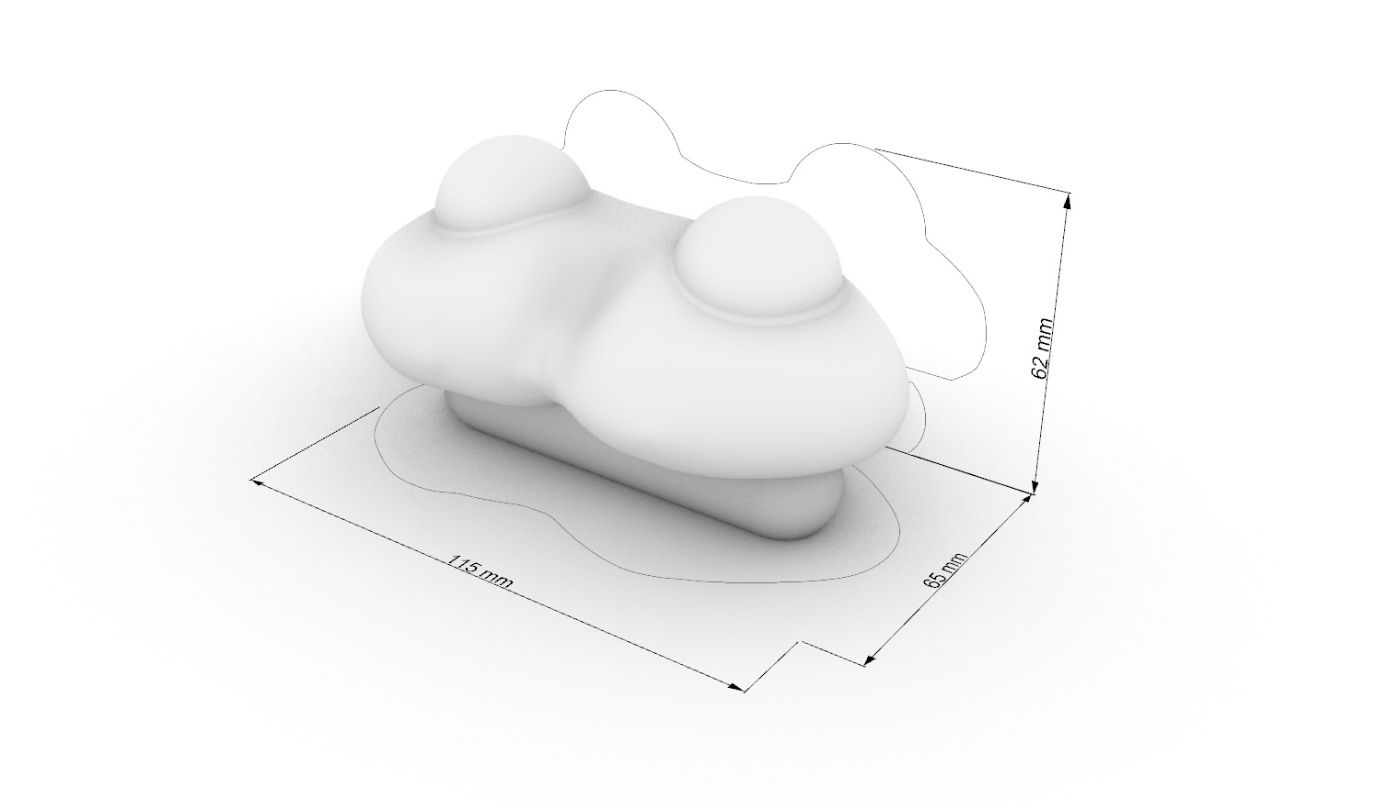


Procedure can be performed using either two needle-holders or one needle-holder and a Maryland or Johan grasper, according to participants preference. A 4-0 Prolene suture mounted on a small round needle is provided before perfusion begins, positioned outside the laparoscopic trainer for accessibility. Participants may select the suture length (recommended 10-15 cm), and the tutor provides guidance upon request.

Once the participant confirms readiness, the tutor fully opens the perfusion line, allowing active bleeding to begin. This marks the start of the timing interval. The participant should promptly identify the site of injury, achieve optimal needle positioning, and perform a continuous or interrupted closure of the defect while minimizing blood loss. The goal is to restore vascular integrity using an atraumatic technique and precise tension control.

Task completion is achieved when the final suture is tied, the needle is removed from the box, and the vessel demonstrates watertightness with no visible leakage under pressure, by opening up the system, for at least 10 seconds. This simulates haemostasis testing in a clinical setting. The end time is recorded when the needle is completely withdrawn from the operative field.

The attempt is terminated and recorded as a failure if simulated blood loss exceeds 3 L. The task therefore provides a controlled yet realistic environment to assess procedural efficiency, technical precision, and the par’s ability to perform effective vascular repair under simulated emergency conditions.
