# Supplementary material for: Consensus-informed Development of Scoring Systems for Intermediate Laparoscopic Simulation Modules: An ESU Laparoscopic Workgroup Initiative
Source: Eur Urol Open Sci. 2026 Apr 15;87:100–6. doi: 10.1016/j.euros.2026.03.014 (PMC13101638; doi:10.1016/j.euros.2026.03.014)
Supplement: Supplementary Data 4 [file mmc6.pdf]

Score Form LUSs2 exam

Name of Examinee **Name of Examiner**

**Task 3 Partial Nephrectomy**<br>Start time: when the trainee starts clamping the artery<br>Stop time: when the surgeon stops the clamping of the artery	**Trial 1**	**Trial 2**<br>(only if trial 1 failed)
Time to complete task:<br>To pass: **…min:sec**	………/……..<br>(Min:sec)	………/……..<br>(Min:sec)
Quality Criteria<br>Rough handling of renal parenchyma (tear in rubber)	OK/not OK	OK/not OK
Quality Criteria<br>Positive surgical margin	OK/not OK	OK/not OK
Quality Criteria<br>>250 ml of blood loss	OK / Not OK	OK / Not OK
Quality Criteria<br>Over time limit of clamping 30 minutes	OK / Not OK	OK / Not OK

Score Form LUSs2 exam

Name of Examinee	Name of Examiner

**Task 2 Major Vessel Injury**<br>Start time: Infusion bag is opened and the bleeding starts<br>Stop time: when suture is cut after a successful suturing and the needle is out of the box.	**<u>Trial 1</u>**	**<u>Trial 2</u>**<br>(only if trial 1 failed)
Time to complete task:<br>To pass: **…min:sec**	………..:…………<br>(Min:sec)	………..:…………<br>(Min:sec)
Quality Criteria<br>Rough handling of tissue resulting in damage to the vessel	YES / NO	YES / NO
Quality Criteria<br>Bleeding is coming out of the injury 10 seconds after stopping time.	OK / Not OK	OK / Not OK
Quality Criteria<br>Blood loss >3 Liters	OK / Not OK	OK / Not OK

Partial Nephrectomy model assessment

Demographics

Your Grade Trainee Tutor/Trainer

Country

Pre-test self-assessment

1. How prepared do you feel to perform a laparoscopic partial nephrectomy (**mainly excision of the tumour and renorraphy)?**
   1 2 3 4 5 6 7 8 9 **10**
   Not prepared at all — Completely prepared to perform independently
2. How much experience in laparoscopic partial nephrectomy have you had in your training thus far?
   1 2 3 4 5 6 7 8 9 **10**
   1 - No experience at all — 10 - Completely trained
3. How many of the following procedures have you performed?
   a. Laparoscopic nephrectomy ……………………
   b. Laparoscopic pyeloplasty……………………….
4. How much will the simulation model help you learn the procedure?
   1 2 3 4 5 6 7 8 9 **10**
   1 - Not at all — 10 - Completely
5. How much do you expect this model to reflect surgery on a live patient?
   1 2 3 4 5 6 7 8 9 **10**
   1 - Not realistic — 10 - Identical to a live patient

Post-test self-assessment

6. Did the tissue's overall appearance match what you would expect during a live surgery?
   1 2 3 4 5 6 7 8 9 **10**
   1 - Not realistic — 10 - Identical to a live patient

7. Rate the following about the model

Consistency	**1** Not realistic	2	3	4	5	6	7	8	9	**10 Very Realistic**
Elasticity	1	2	3	4	5	6	7	8	9	10
Turgor	1	2	3	4	5	6	7	8	9	10
Feel	1	2	3	4	5	6	7	8	9	10
Appearance of the kidney	1	2	3	4	5	6	7	8	9	10
Appearance of the tumour	1	2	3	4	5	6	7	8	9	10

8. Did your ability to suture the model match your expectations as in a live patient?
   1 2 3 4 5 6 7 8 9 **10**
   1 - Does not match my expectations at all **10** - Completely matches my expectations

9. Did you feel the same pressure in this environment with this model?
   1 2 3 4 5 6 7 8 9 **10**
   1 - Not realistic 10 - Very realistic

10. Do you feel more practice with this model will help you learn and refine the surgical operation?
    1 2 3 4 5 6 7 8 9 **10**
    1 - Strongly disagree 10 - Strongly agree

Do you have any free comments about this model?

- ..
- ..
- ..
