# Supplementary material for: Consensus-informed Development of Scoring Systems for Intermediate Laparoscopic Simulation Modules: An ESU Laparoscopic Workgroup Initiative
Source: Eur Urol Open Sci. 2026 Apr 15;87:100–6. doi: 10.1016/j.euros.2026.03.014 (PMC13101638; doi:10.1016/j.euros.2026.03.014)
Supplement: Supplementary Data 5 [file mmc7.docx]

**Table 4_ Partial Nephrectomy Do’s and Don’ts**

| **Do** | **Don´t** |
| --- | --- |
| Complete the procedure within the defined time limit (≤40 minutes) | Exceed the benchmark operative time (>40 minutes) |
| Perform a complete tumour enucleation | Leave positive surgical margins (incomplete enucleation) |
| Handle tissues gently and precisely. | Rough tissue handling, with possible tissue laceration or damage |
| Appropriate suturing technique | Neglect proper renorrhaphy technique |
