# Supplementary material for: Consensus-informed Development of Scoring Systems for Intermediate Laparoscopic Simulation Modules: An ESU Laparoscopic Workgroup Initiative
Source: Eur Urol Open Sci. 2026 Apr 15;87:100–6. doi: 10.1016/j.euros.2026.03.014 (PMC13101638; doi:10.1016/j.euros.2026.03.014)
Supplement: Supplementary Data 6 [file mmc8.docx]

**Table 5_ Major Vessel Injury Do’s and Don’ts**

| **Do** | **Don´t** |
| --- | --- |
| Complete the task within the defined time limit | Over the time limit |
| Ensure minimal blood loss | Blood loss >3L |
| Use gentle tissue handling | Rough handling of the vessel (tear in the rubber) |
| Select appropriate instruments and suture length | Additional damage to the vessel |
| Achieve haemostasis so that there is no visible bleeding and vessel is properly sealed | Bleeding is coming out of the injury 10 mins after the stop time |
